# Recurrent 8q13.2-13.3 microdeletions associated with Branchio-oto-renal syndrome are mediated by human endogenous retroviral (HERV) sequence blocks

**DOI:** 10.1186/s12881-014-0090-9

**Published:** 2014-08-19

**Authors:** Xiaoli Chen, Jun Wang, Elyse Mitchell, Jin Guo, Liwen Wang, Yu Zhang, Jennelle C Hodge, Yiping Shen

**Affiliations:** 1 Beijing Municipal Key Laboratory of Child Development and Nutriomics, Capital Institute of Pediatrics, Beijing, China; 2 Department of Neurology, Affiliated Children’s Hospital of Capital Institute of Pediatrics, Beijing, China; 3 Department of Laboratory Medicine and Pathology, Mayo Clinic, Rochester, MN, USA; 4 Department of Pathology and Laboratory Medicine, Cedars-Sinai Medical Center, Los Angeles, CA, USA; 5 Shanghai Children’s Medical Center, Shanghai Jiaotong University School of Medicine, Shanghai, China; 6 Department of Pathology, Harvard Medical School, Boston, MA, USA; 7 Department of Laboratory Medicine, Children’s Hospital Boston, Boston, MA, USA

**Keywords:** De novo 8q13.2-13.3 microdeletion, Human endogenous retroviral (HERV) sequences, Branchio-oto-renal syndrome, Mesomelia-synostoses syndrome

## Abstract

**Background:**

Human endogenous retroviral (HERV) sequences are the remnants of ancient retroviral infection and comprise approximately 8% of the human genome. The high abundance and interspersed nature of homologous HERV sequences make them ideal substrates for genomic rearrangements. A role for HERV sequences in mediating human disease-associated rearrangement has been reported but is likely currently underappreciated.

**Methods and Results:**

In the present study, two independent *de novo* 8q13.2-13.3 microdeletion events were identified in patients with clinical features of Branchio-Oto-Renal (BOR) syndrome. Nucleotide-level mapping demonstrated the identical breakpoints, suggesting a recurrent microdeletion including multiple genes such as *EYA1, SULF1,* and *SLCO5A1*, which is mediated by HERV1 homologous sequences.

**Conclusions:**

These findings raise the potential that HERV sequences may more commonly underlie recombination of dosage sensitive regions associated with recurrent syndromes.

## Background

Low-copy repeat (LCR) or segmental duplication (SD)-mediated non-allelic homologous recombination (NAHR) is a common mechanism that results in microdeletions or microduplications [1]. Many of these rearrangements are responsible for human genomic disorders [2],[3]. Typically SDs range from 10 to hundreds of kilobases with >95% sequence identity and are involved in chromosomal recombination [4]; this usually occurs in a constrained footprint [5]. Indeed, smaller interspersed repeats like LINE, Alu, and human endogenous retrovirus (HERV) elements have also been shown to mediate recurrent genomic rearrangements [5],[6]. For example, whole genome analysis showed that >16% of HERV-K elements have undergone rearrangements that result in larger-scale deletion/duplication and chromosome reshuffling in primate genomes [7]. In addition, homologous recombination between HERV15 elements on the Y chromosome mediates a recurrent Y chromosome microdeletion which removes the azoospermia factor a (AZFa) region that results in male infertility [8],[9]. Finally, a recurrent translocation between 4q35.1 and 18q22.3 mediated by HERV-H recombination also has been reported recently [10].

Branchio-oto-renal syndrome (BOR, OMIM 113650) is an autosomal dominant disorder characterized by sensorineural, conductive, or mixed hearing loss, structural defects of the outer, middle, and inner ear, branchial fistulas or cysts, and renal abnormalities ranging from mild hypoplasia to complete absence [11]. The estimated prevalence of BOR syndrome is 1:40,000 and it affects approximately 2% of profoundly deaf children [12],[13]. This syndrome is clinically and genetically heterogeneous and has a high penetrance with variable expressivity [11],[13]. Mutations on the human ortholog of the Drosophila eyes absent gene *(EYA1*, OMIM 601653) are considered to be a major cause of BOR syndrome. Approximately 40% of BOR patients have mutations in the *EYA1* gene; 117 different heterozygous pathogenic variants including frame shift, stop, splice-site, and missense mutations have been reported [14]-[18]. Large heterozygous deletions at 8q13.2-13.3 encompassing the *EYA1* gene have also been detected in patients with BOR [19]-[21]. Here we report two independent *de novo* 8q13.2-13.3 microdeletion events in two patients with clinical features of BOR. We provide evidence that this microdeletion is recurrent and mediated by HERV1 homologous sequences. Given that HERV sequences comprise approximately 8% of the human genome, our findings support the potential for these sequences to have a broader role in mediating recurrent disease-associated recombinations and polymorphic rearrangements on a genome-wide scale.

## Methods

The Capital Institute of Pediatrics Review Board and the Mayo Clinic Institutional Review Board approved this project. The Written informed consent was obtained from the patient’s guardian/parent/next of kin for the publication of this report and any accompanying images.

The physical and neurological examinations by a developmental specialist as well as biochemical and other medical evaluations were completed on case 1 at the Affiliated Children’s Hospital of Capital Institute of Pediatrics. IQ was measured by Wechsler Intelligence Scale for Children (WISC). Phenotype information regarding case 2 was supplied by the Mayo Clinic Cytogenetics Laboratory at the time of clinical chromosomal microarray testing.

DNA from peripheral blood was isolated by the Blood and Tissue kit (Qiagen, Valencia, CA). Array CGH was performed using Agilent 244 K and 180 K oligonucleotide platform (Agilent Technologies Inc., Palo Alto, CA) for case 1 and 2, respectively.

Long-range PCR (Platinum PCR SuperMix High Fidelity kit, Invitrogen, Carlsbad, CA) was performed to locate the junction regions using multiple breakpoint-specific primers around the approximate breakpoints (Additional file 1: Table S1). The nested PCR was performed to confirm the deletion breakpoints in the two independent deletion cases. During the nested PCR, a 6.5 kb fragment outside two HERV blocks was first amplified from the long-range PCR products using the junction primers (1F and 1R in Additional file 1: Table S1) [22]. The 6.5 kb fragment was then purified from agarose gels using the QIAquick gel extraction kit (Qiagen, Valencia, CA) and diluted 1:10 to be used as the template for the second-round PCR (primers 2F and 2R in Additional file 1: Table S1). The junction fragment was, visualized on a 1% agarose gel, purified using Exonuclease I (New England Biolabs Inc., MA, USA), and then cloned into a TOPO-TA vector for Sanger sequencing following the manufacturer’s protocol. A mixture of DNA from 10 normal children was used as the control.

## Results

### Clinical reports

**Figure 1:**
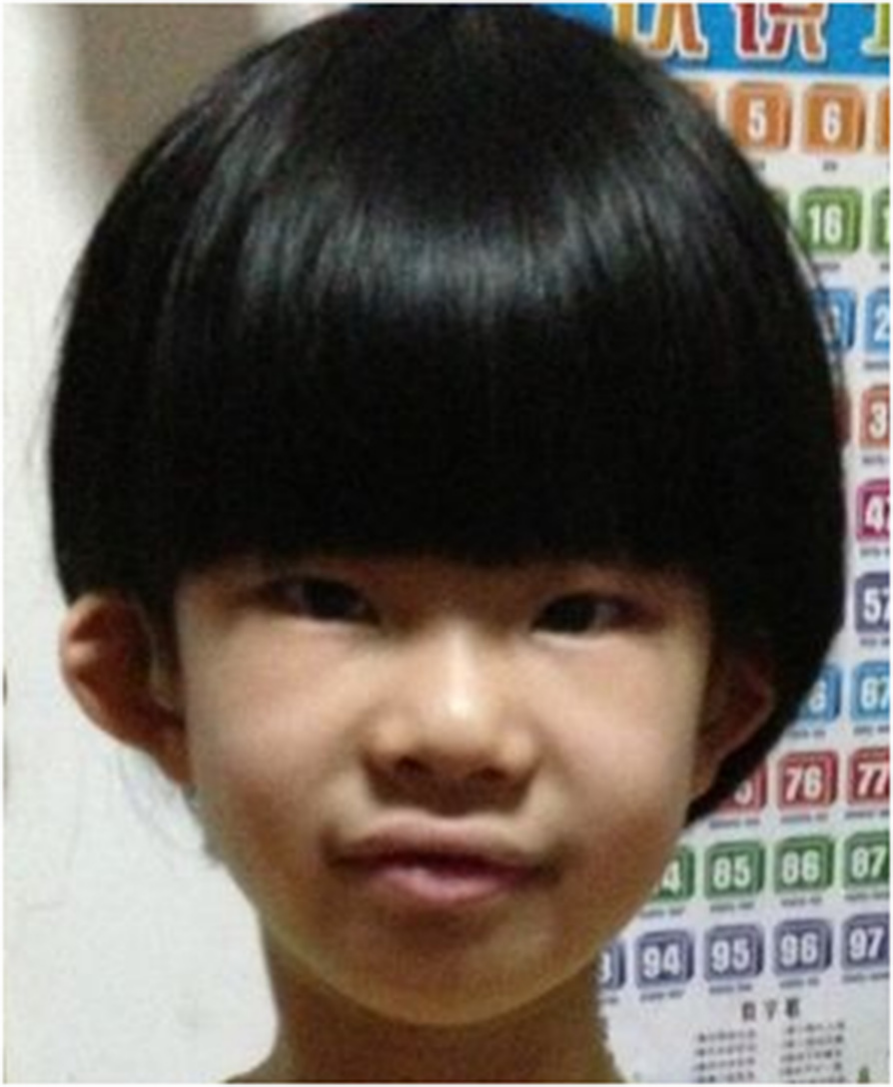
**The dysmorphic facial features of case 1 with** ***de novo*** **8q13.2-13. microdeletion.** Low-set ears with an asymmetrical, cup-shape on the left and preauricular pits near the right ear (black arrow), and bilateral hearing loss.

Case 1 is a Chinese girl born to unrelated parents. Prenatal development was normal except for ultrasound detection of a cyst in the left kidney at 35 gestational weeks. She was born at 40 gestational weeks by Cesarean section with a birth weight of 4 kg and a birth length of 50 cm. The Apgar scores were normal at both one and five minutes after birth. Abnormal hearing was noticed at newborn hearing screening. At three months of age, an initial CT scan diagnosed bilateral cochlear dysplasia with incompletely formed cochlea and posterior semicircular. At two years old, an auditory brainstem response (ABR) test confirmed moderate conductive hearing loss in both ears. On physical examination at four years of age, she was 110 cm (97th centile) and 16 kg (25-50th centile). The renal ultrasound showed bilateral hydronephrosis/pyelectasis. A routine urine test was negative and the levels of serum urea nitrogen (3.1umol/L, normal range is 2.50-8.21 mmol/L) and creatinine (53.2umol/L, normal range is 53–114.9umol/L) were consistent with normal renal function. Her second temporal CT scan performed at the age of two years showed bilateral inner ear malformations with cochlear hypoplasia (approximately 2 turns), incompletely formed lateral semicircular canal, enlargement of vestibular window and dilation of the right vestibular aqueduct. The images also manifested deep middle fossa. Her motor and neurological developmental skills were normal (IQ = 83) with the exception of language delay (vocabulary developmental index = 67) due to hearing loss. The dysmorphic features included high arched palate, branchial anomalies and a preauricular pit, and a cup-shape to the left low-set ear (Figure 1). The karyotype was normal 46, XX.

Case 2 is a two months old Middle Eastern male baby born to parents who are not documented to be related. Medical exam showed short stature, dysmorphic features including macrocephaly, malformed and low set ears, high arched palate, short neck, wide spaced eyes, and flat occiput. The karyotype was 46, XY. He was noted to have ear anomalies, although whether he has hearing loss is unknown based on the available information in his medical record.

### Array-CGH

**Figure 2:**
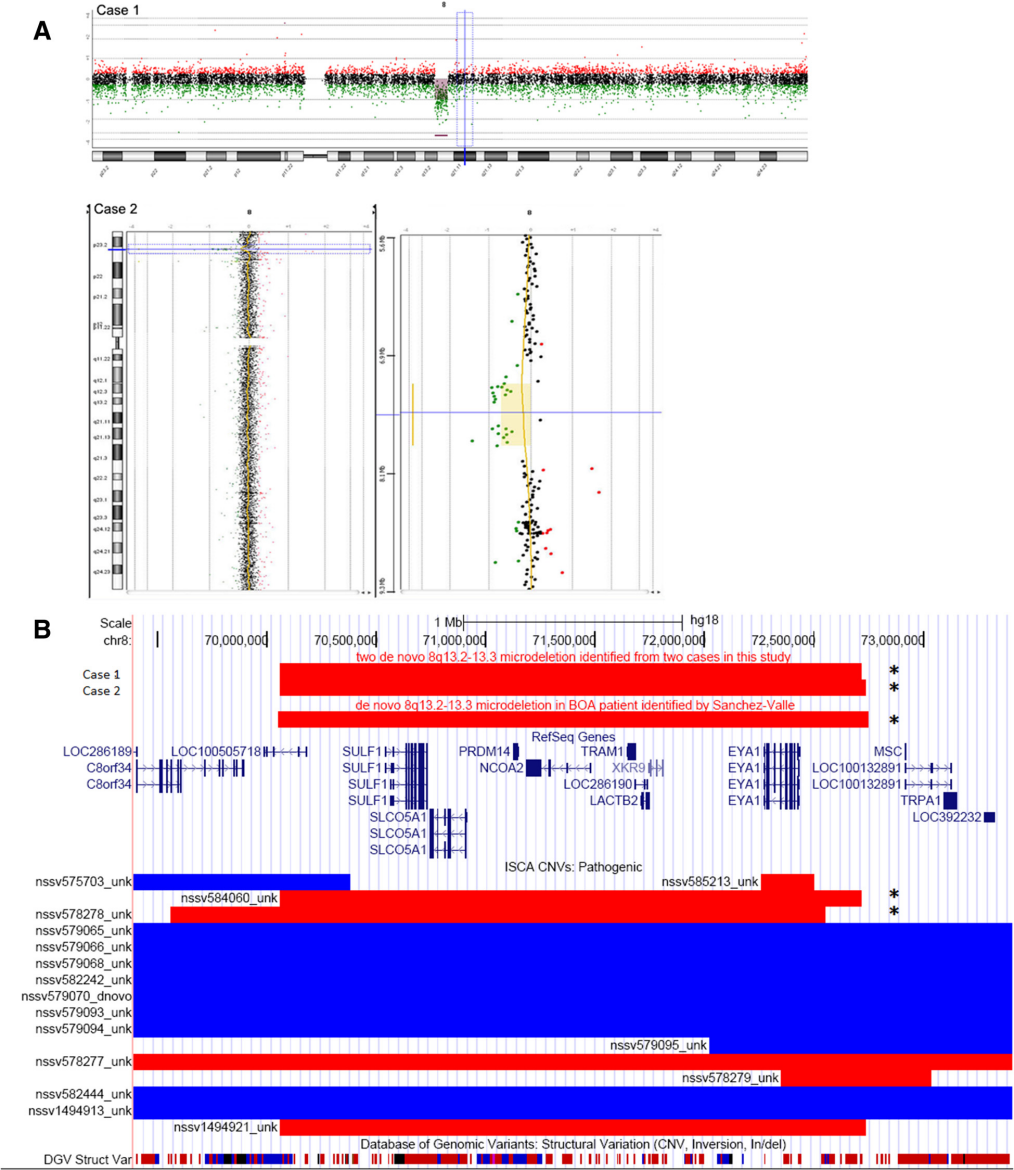
**Recurrent,** ***de novo*** **8q13.2-13.3 microdeletion identified in four cases with Branchio-oto-renal syndrome. A** Array data plots showing 2.6 Mb deletions ranging from 70,062,624-72,714,795 at 8q13.2-13.3 in cases 1 and 2. **B** The UCSC Genome Browser custom tracks (chr8:69,393,700-73,407,100, genome build hg18) demonstrating deletion of 8q13.2-13.3 in five unrelated cases with BOR syndrome/hearing problems (labeled as *), which cover 10 Refseq genes (*LOC100505718, SULF1, SLCO5A1, PRDM14, NCOA2, TRAM1, LOC286190, LACTB2, XKR9, EYA1*). Red indicates deletion and blue indicates duplication. Note: ns1492921 in the ISCA data represents the same case as case 2 in this study.

A 2.6 Mb microdeletion located at 8q13.2-13.3 was detected in both case 1 (chr8:70,062,624-72,714,795 in genome build hg18) and case 2 (chr8:70,062,624-72,738,255 in genome build hg18, Figure 2A). Parental testing confirmed they were both *de novo* deletions; array CGH and real-time quantitative PCR were employed in case 1 while FISH (BAC probe RP11-744 N15) was used for case 2. There are ten Refseq genes (*LOC100505718, SULF1, SLCO5A1, PRDM14, NCOA2, TRAM1, LOC286190, LACTB2, XKR9, EYA1*, Figure 2B) in this region and six genes reported in OMIM (*SULF1, SLCO5A1, PRDM14, NCOA2, TRAM1, EYA1*). In addition, one BOR patient reported by Sanchez-Valle et al. (chr8:70,053,688-72,748,049 in genome build hg18) [22], and two cases in The International Standards for Cytogenomic Arrays Consortium (ISCA) dataset (http://www.iccg.org/; nssv578278 at chr8:69,400,250-72,388,412 and nssv584060 at chr8:70,062,670-72,714,817) had a similar size (Figure 2B). It is of note that another case in the ISCA dataset, ns1494921, is the same case as case 2 in our study. All five independent cases share similar breakpoints (labelled * in Figure 2B), suggesting the recurrent nature of this deletion.

### Breakpoint mapping

**Figure 3:**
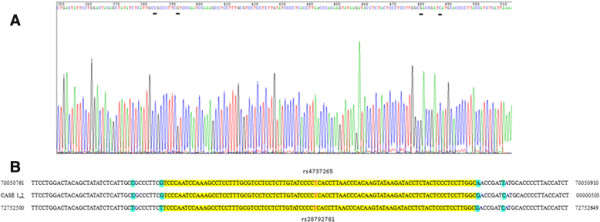
**Nucleotide-level mapping of the recurrent 8q13.2-13.3 deletions.A** Schematic figure of mapping primers used in long-range and nested PCR. The numbers are the coordinate of chromosome 8 (gnome build hg18). HERVH are presented as two red rectangles (HERVH-int1 and HERVH-int2). The black lines represent the genomic size of the 8q13 region. The green and blue lines represent the size of the amplicons produced by the long-range and nested PCR, respectively. The small brown and blue dots in the red rectangles represent the primers 2F and 2R, and the larger black dots represent the primers 1F and 1R. While primer 1F and 1R, 2R are unique sequences in the human genome, the 2F is scattered sequence in the human genome but is unique for the amplicon produced by the nested PCR. The 800 bp fragment produced in the nested PCR was also amplified in the control due to two identical sequences of the 2F primer in HERVH-int1 and HERVH-int2 (two brown dots in two HERVH blocks). **B** Gel electrophoresis images (0.8% agarose) of PCR products from the two cases and a control. P1: case 1, P2: case 2, C: mixed DNA from 10 controls. Lane 1–2: DNA marker ladder (M); Lane 3–5: long-range PCR products using genomic DNA with primer 1F and 1R; Lane 7–9: general PCR products using genomic DNA with primer 2F and 2R; Lane 11–12: nested PCR products using the amplicon in lanes 3 (P1-1) and 4 (P2-1) as template with primers 2F and 2R; Lane 6 and 10: blanks.

The breakpoint-specific primer pair (BP-3 F and BP-3R in Additional file 1: Table S1) revealed an identical 8.5 kb amplicon in both case 1 and 2 which was not present in the control. To demonstrate the recurrent nature of the deletions and to understand their molecular mechanism, the junction region was mapped using long-range PCR and nested PCR design (The locations of the primers are shown in Figure 3A). Using the primer pair described in Sanchez-Valle et al. (1F and 1R in Additional file 1: Table S1, black dots in Figure 3A), an identical 6.5 kb junction fragment was also amplified in both cases (lane 3 and 4 in Figure 3B). This fragment is the same size as what was amplified in patient 1 in the report by Sanchez-Valle et al. [22]. Similarly, no amplification was detected for mixed control DNA. Thus, the junction sequences are deletion specific and two patients share similar breakpoints.

**Figure 4:**
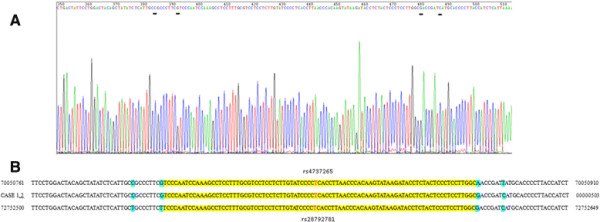
**The sequence characteristics of the breakpoint junction in the recurrent 8q13.2-13.3 deletion. A** The raw sequence data from the 800 bp-size breakpoint junction. The black lines beneath the base calls represent four locus-specific SNPs used as landmarks in the breakpoint identification. First two SNPs (C and G) are located in HERVH-int1 and the latter two SNPs (G and C) are located in HERVH-int 2. **B** Alignment of the junction sequences from case 1 and 2. BLAST was used to compare the partial sequences of two HERV blocks (chr8:70,050,761-70,050,910, chr8:70,2752,500-72,752,649) with the 800 bp of nested PCR product. The four locus-specific SNP that differentiate the HERV blocks are highlighted in blue while two common SNPs are shown in red. The 87 bp recombination region is highlighted in yellow.

The nested PCR was performed (2F and 2R in Additional file 1: Table S1, brown and blue dots in Figure 3A) using the 6.5 kb junction sequence as a template. An 800 bp fragment was amplified from both cases (lane 11, 12 in Figure 3B). The sequences of the primer 2F is not unique in the human genome, and so an 800 bp product was generated in the control as expected when genomic DNA was used as the template (lane 7–9 in Figure 3B). The sequence of the 800 bp product revealed that two human endogenous retroviral sequence blocks (HERVH-int1, chr8:70,048,221-70,051,505 and HERVH-int2, chr8:72,750,179-72,753,246, identify is >95%, Figure 3A) are involved in this deletion. Using four locus-specific SNPs of the two HER regions (labeled black in Figure 4A, highlighted blue in Figure 4B) and two common SNPs (rs4737265 and rs28792781, red font in Figure 4B) as landmarks, the deletion breakpoints were narrowed to two 86 bp intervals (highlighted yellow in Figure 4B) which are identical between the two adjacent HERV blocks (chr8:70,050,798-70,050,884 and chr8:72,752,537-72,752,623).

## Discussion

Human Endogenous Retroviral (HERV) sequences, which are remnants of past retroviral infection, make up approximately 8% of the human genome [23]. The interspersed nature and high sequence homology of HERVs make them ideal substrates for NAHR in the human genome [24]. In fact, greater than 16% of human HERV-K elements may have mediated large-scale genome rearrangements during primate evolution [7], and inverted HERV-K elements are known to cause the frequent 8p23.1 polymorphic inversions in humans [25]. It is known that homologous recombination between distant HERVs can cause inherited male infertility due to loss of the 792-kb fragment encompassing azoospermia factor a (AZFa) region [8],[9]. Sanchez-Valle et al. reported the first BOR patient with 8q13.2-13.3deletion and proposed that this microdeletion was mediated by two adjacent HERV blocks [22]. A genome-wide copy number variant analysis was performed for a Branchio-oto-renal syndrome cohort with negative pathogenic mutation in known BOR-causing genes [26]. Four carriers with 8q13.2-13.3 deletions (2.7 Mb deletion, chr8:70053668–72748108) were identified that share approximatly identical breakpoints covering HERV blocks, proving 8q13.2-13.3 deletion is a recurrent genomic rearrangement event.

In the current study, we mapped the breakpoint of 8q13.2-13.3 deletions in two unique cases, and found the breaks occurred in identical 86 bp homologous sequence within HERV regions. We therefore demonstrated that 8q13.2-13.3 deletions associated with BOR syndrome features can be recurrent through an NAHR mechanism, which places this microdeletion syndrome into the same category as DiGeorge syndrome and William’s syndrome. Similarly, HERV sequences were reported to mediate recurrent 3q13.2-q13.31 deletions which cause a new syndrome of hypotonia and motor, language, and cognitive delays [27]. Beside such inter-chromosomal arrangements, 150 bp homologous sequences of two HERV-H blocks have been shown to mediate a recurrent intra-chromosomal translocation between 4q and 18q [10]. The above published studies and our results suggest that HERV regions can induce both inter-chromosomal and intra-chromosomal rearrangements.

8q13.2-13.3 microdeletion has been associated with several medical disorders due to haploinsufficiency of genes within the region. Deletion of *EYA1* is associated with BOR syndrome [11],[19],[20],[28]. The typical manifestations of BOR are hearing loss and structural defects of the outer, middle, and inner ear (98.5%), preauricular pits (83.6%), branchial anomalies (68.5%), renal anomalies (38.2%), and external ear abnormalities (31.5%) [11]. 8q13 has also reportedly been associated with Mesomelia-Synostoses syndrome (MSS, OMIM 600383) due to the co-deletion of *SULF1* and *SLCO5A1*[29]. MSS is a rare dominantly inherited disorder characterized by mesomelic limb shortening and acral synostoses, renal malformations and/or congenital heart defects occur sporadically. Hearing loss has not been reported in MSS patients [30],[31], although <10 patients with MSS have been described [29],[32]. We summarized the clinical phenotypes of reported carriers with 8q13.2-13.3 microdeletion carriers reported in the literatures (Table 1) [22],[26]. Neither mesomelic short stature nor severe skeletal changes was observed. Their predominant clinical features include hearing loss (4/6), branchial fistulae (5/6), preauricular pits (4/6) and hydronephrosis/kidney agenesis (4/6), which are major suggestive phenotypes of BOR syndrome. Our literature review suggests that recurrent 8q13.2-13.3 deletion is an important cause of BOR. However, not all BOR cases result from recurrent 8q13.2-13.3 deletion by a HERV mediated non-allelic homologous recombination mechanism. Some BOA patients have non-recurrent alternations, such as the *EYA1* exonic deletion, exemplified by case nssv525813 in the ISCA dataset (Figure 2B). Based on published diagnostic scoring criteria for BOR [16], case 1 in this study and four patients in the literatures meet the definition of typical BOR (hearing loss and preauricular pits, inner ear and renal anomalies), while case 2 in this study represents atypical BOR (ear anomalies or ear symptoms). Thus the results are consistent with phenotype heterogeneity of the 8q13.2-13.3 deletion. The functional polymorphism of the *EYA1* hemizygous allele or another dosage sensitive gene in the deleted region could potentially be responsible for the phenotype heterogeneity.

**Table 1:** Clinical findings in six patients with recurrent 8q13.2-13.3 deletion

		**Case 1 in this study**	**Case 2 in this study**	Patient 1*	21230**	20960**	518240**
**Major BOR criteria**	Hearing loss/impairment	+	NA	+	-	+	+
	Branchial fistulae	+	NA	+	+	+	+
	Preauricular pits	+	NA	+	+	-	+
	Hydronephrosis/kidney agenesis	+	NA	+	+	-	+
**Minor BOR criteria**	Ear anomalies or ear symptoms (cup ear, low-set ear, asymmetry ear)	+	+	+	+	+	-
**Dysmorphic facial features**		High arched palate	Macrocephaly, high arched palate, short neck, wide spaced eyes, and flat occiput	Left-sided microphthalmos with iris coloboma	Small mouth, branchial tag	-	-
**Non-BOR related abnormalities**	Intellectual disability/developmental delay	-	NA	+	-	-	-
	Speech/language delay	+	NA	+	-	-	-
	Short stature	-	Proportional short stature	Proportional short stature	-	-	-

Only patient with detailed clinical information was recruited; +: present; −: absent; NA: not available in the medical record; *Reported by Sanchez-Valle et al.; **Three patients reported by Brophy PD et al., one patient’s (CDS2893) clinical information wasn’t available.

## Conclusion

In summary, we confirmed in two cases the recurrent nature of two 8q13.2-13.3 microdeletion associated with BOR syndrome. We also mapped the deletion breakpoints and demonstrated that the deletion is mediated by two adjacent HERVH sequences. Therefore, this microdeletion represents another example of HERV-mediated NAHR, indicating a more prominent role for the HERV regions in recurrent genomic disorders.

## Competing interests

The authors declare that they have no competing interests.

## Authors’ contributions

XLC and YPS designed the study, performed the data analysis and manuscript writing. XLC contributed to long-range and nested PCR; JW and LWW helped in patient recruitment and clinical checkup and obtaining a genetics questionnaire; JG performed DNA extraction and an array experiment; YZ performed the clone and gel purification and sequencing; EM and JH helped in patient recruitment and contributed to manuscript writing. All authors read and approved the final manuscript.

## Additional file

## Supplementary Material

Additional file 1: Table S1. Primers used for long-range PCR and nested PCR. Click here for file
