# Supplementary material for: Recurrent 8q13.2-13.3 microdeletions associated with Branchio-oto-renal syndrome are mediated by human endogenous retroviral (HERV) sequence blocks
Source: BMC Med Genet. 2014 Aug 19;15:90. doi: 10.1186/s12881-014-0090-9 (PMC4152767; doi:10.1186/s12881-014-0090-9)
Supplement: Additional file 1: Table S1. — Primers used for long-range PCR and nested PCR. [file s12881-014-0090-9-S1.doc]

**Additional file 1:**

**Table S1 Primers used for long-range PCR and nested PCR**

|  | **Primer name** | **Primer sequence (5’→3’)** | **Product length (bp)** |
| --- | --- | --- | --- |
| **Long-range PCR** | BP-1F | TCCACAATGCCACAGGAGTA |  |
|  | BP-1R | AGCCTGGTGATGGCAGATAA | **12kb** |
|  | BP-2F | CAGCCTTCCCTTGGTGTTTA |  |
|  | BP-2R | ATGAACCCAAGTGCAAGACC | **10kb** |
|  | BP-3F | CCCAAGGCTCTCTGACTGAC |  |
|  | BP-3R | GAGTGGCAGTTTGGGGATAA | **8kb** |
| **Nested PCR** | **1F*** | CCGTAGCCCTGAATGTTGACTACTATACCC |  |
|  | **1R*** | AACAATACGGTGACTGAAAGATGTGTGTTG | **6.5kb** |
|  | **2F** | CCCAAGGCTCTCTGACTGAC |  |
|  | **2R** | TGGGGGAAATGTAAGGAAAG | **800bp** |
|  |  |  |  |
|  |  |  |  |

* primers from report of Sanchez-Valle A;
